# Effect of Dietary Cocoa Tea (*Camellia ptilophylla*) Supplementation on High-Fat Diet-Induced Obesity, Hepatic Steatosis, and Hyperlipidemia in Mice

**DOI:** 10.1155/2013/783860

**Published:** 2013-07-09

**Authors:** Xiao Rong Yang, Elaine Wat, Yan Ping Wang, Chun Hay Ko, Chi Man Koon, Wing Sum Siu, Si Gao, David Wing Shing Cheung, Clara Bik San Lau, Chuang Xing Ye, Ping Chung Leung

**Affiliations:** ^1^College of Chemistry and Biological Science, Yili Normal University, Yining, Xinjiang 835000, China; ^2^Department of Biology, School of Life Science, Sun Yat-sen University, Guangzhou 510275, China; ^3^Institute of Chinese Medicine, The Chinese University of Hong Kong, Shatin, New Territories, Hong Kong; ^4^State Key Laboratory of Phytochemistry and Plant Resources in West China, The Chinese University of Hong Kong, Shatin, New Territories, Hong Kong; ^5^The Chinese University of Hong Kong Shenzhen Research Institute, Shenzhen 518057, China

## Abstract

Recent studies suggested that green tea has the potential to protect against diet-induced obesity. The presence of caffeine within green tea has caused limitations. Cocoa tea (*Camellia ptilophylla*) is a naturally decaffeinated tea plant. To determine whether cocoa tea supplementation results in an improvement in high-fat diet-induced obesity, hyperlipidemia and hepatic steatosis, and whether such effects would be comparable to those of green tea extract, we studied six groups (*n* = 10) of C57BL/6 mice that were fed with (1) normal chow (N); (2) high-fat diet (21% butterfat + 0.15% cholesterol, wt/wt) (HF); (3) a high-fat diet supplemented with 2% green tea extract (HFLG); (4) a high-fat diet supplemented with 4% green tea extract (HFHG); (5) a high-fat diet supplemented with 2% cocoa tea extract (HFLC); and (6) a high-fat diet supplemented with 4% cocoa tea extract (HFHC). From the results, 2% and 4% dietary cocoa tea supplementation caused a dose-dependent decrease in (a) body weight, (b) fat pad mass, (c) liver weight, (d) total liver lipid, (e) liver triglyceride and cholesterol, and (f) plasma lipids (triglyceride and cholesterol). These data indicate that dietary cocoa tea, being naturally decaffeinated, has a beneficial effect on high-fat diet-induced obesity, hepatomegaly, hepatic steatosis, and elevated plasma lipid levels in mice, which are comparable to green tea. The present findings have provided the proof of concept that dietary cocoa tea might be of therapeutic value and could therefore provide a safer and cost effective option for patients with diet-induced metabolic syndrome.

## 1. Introduction

 Metabolic syndrome refers to the clusters of risk factors that would lead to increased episodes of cardiovascular disease (CVD). These risk factors include age, family history of CVD, gender, obesity, insulin resistance, nonalcoholic fatty liver (NAFLD), dyslipidemia, hypertension, and type 2 diabetes [1, 2]. According to the American Association of Clinical Endocrinologists and the International Diabetes Federation (IDF), metabolic syndrome can be defined as a complex of symptoms with central obesity (with waist circumference of >102 cm in men or >88 cm in women), plus two or more of the following factors: elevated serum levels of triglycerides, hyperglycemia, elevated blood pressure, and reduced serum levels of high-density lipoprotein- (HDL-) associated cholesterol [3]. 

Because of the current epidemic of over-nutrition and sedentary lifestyle worldwide, metabolic syndrome is becoming more common, particularly in developed countries. According to the report from the National Cholesterol Education Program (NCEP), the prevalence of metabolic syndrome is high, with a rate of 35.1% for middle-aged men and 32.6% for middle-aged women in the United States [3, 4]. However, since the pathogenesis of metabolic syndrome has multiple metabolic origins, pharmacological approaches often consist of separate drugs which target at individual risk factors: lipid-lowering drugs, antihypertensive agents, hypoglycemic drugs, antiplatelet drugs, and weight-loss agents [5, 6]. These drugs nonetheless pose various side effects risks. Functional foods or nutraceuticals which have potentially important antiobesity properties have thus attracted great attention. 

Tea (*Camellia sinensis*) is one of the most consumed beverages in the world, second only to water [7, 8]. Depending on the fermentation process, tea can be classified into three main types: the “nonfermented” green tea, the “semifermented” oolong tea, and the “fully fermented” black/red tea [9]. Due to the differences in processing, the chemical compositions of tea also differ, leading to the differences in the biological properties of different types of tea extracts [8]. Whereas the fully fermented black tea contains theaflavins and thearubigins as products from catechins oxidation, green tea has only undergone minimal fermentation and therefore contains predominately catechins [10]. It is estimated that a typical brewed green tea beverage (2.5 g green tea in 250 mL hot water) usually contains approximately 240–320 mg catechins including (−)-epicatechin (EC), (−)-epigallocatechin (EGC), (−)-epicatechin-3-gallate (ECG), epigallocatechin-3-gallate (EGCG), (+)-catechin (C), and (+)-gallocatechin (GC) [10–12]. EGCG is the most abundant catechin present in green tea comprising approximately 30–50% of the total catechins content [13]. In addition to the catechin constituents, green tea also contains considerably high amounts of caffeine (3–6%) [11, 12, 14]. Although it has been well documented that green tea consumption could exhibit antiobesity, hypolipidemic, and hypoglycaemic properties and thereby improving CVD health, effects of caffeine contents within green tea on CVD have been contradictive [15, 16]. The various negative effects that caffeine poses on human behaviours [17] and sleep deprivation [18] have also caused great dilemma to obese or diabetic individuals. There is thus an urge for the market to search for decaffeinated green tea which could pose similar health benefits [19]. 

Cocoa tea (*Camellia ptilophylla*), which belongs to the genus *Camellia*, is a naturally decaffeinated tea plant. For many years, it has been widely consumed by local inhabitants in the Longmen area of Guangdong Province of China but has only started attracting scientific interest since 1988 [20, 21]. Early work suggested that cocoa tea exhibits profound cytotoxic effect on various cancer cell lines including HeLa, CNE2, and MGC-803 [22]. Peng et al. also demonstrated the potential of cocoa tea extract to inhibit prostate cancer cell proliferation and tumour growth *in vivo* and *in vitro* [23]. More recently, our laboratory showed that administration of cocoa tea to HepG2 xenograft nude mice resulted in dose-dependent reduction in tumour growth via caspase-3-regulated pathway [24]. Despite having high catechins content, the beneficial effects of cocoa tea on cardiovascular health have not been studied in details. Kurihara et al. demonstrated that a single oral administration of 500 mg/kg of cocoa tea extract significantly suppressed plasma triglyceride (TG) induction in mice given 5 mL/kg of lard oil [25]. Although this experiment suggested the potential of cocoa tea extract to reduce plasma TG, this effect is acute and the chronic effect of cocoa tea remains to be determined. Furthermore, there exists no experimental data on the effects of cocoa tea on diet-induced obesity and NAFLD. In order to determine whether cocoa tea supplementation to a high-fat diet results in an improvement in obesity and plasma and liver lipid levels, we carried out the present study in C57BL/6 mice to compare the effects of different doses of cocoa tea and green tea supplementations on high-fat diet-induced metabolic syndrome.

## 2. Materials and Methods

### 2.1. Herbal Materials Preparation

Green tea leaves were purchased from renowned supplier in China. Cocoa tea leaves were obtained from the Tea Research Institute, Guangdong Academy of Agricultural Sciences, China. Herbarium voucher specimens of the green tea and cocoa tea were deposited at the museum of the Institute of Chinese Medicine, the Chinese University of Hong Kong, with voucher specimen numbers 3336 and 3401, respectively. For the extracts preparation, green tea or cocoa tea leaves (100 g) were brewed 3 times, each with 1 L of hot distilled water (80°C) for 15 min. The infusion was then cooled to room temperature and filtered with cellulose filter paper (0.45 *μ*m, Millipore, Billerica, MA, USA). The filtrate was then concentrated using a vacuum rotary evaporator. The resulting extracts were freeze-dried overnight to produce tea powder. 

### 2.2. HPLC Analysis of Tea Aqueous Extract

High-performance liquid chromatography (HPLC) analysis was performed using Hewlett Packard Agilent 1100 series HPLC System, equipped with G1329A ALS Autosampler and G1315A Diode Array Detector (Agilent Technologies, USA). Sample solution was injected onto a Supelco Discovery RP Amide C16 guard column (15 cm × 4.6 mm, 5 *μ*m) (Sigma-Aldrich, Inc., USA). All solvents were prefiltered with 0.45 *μ*m Millipore filter disk (Millipore, MA, USA) and degassed. A gradient elution was carried out using the following solvent systems: mobile phase A: double distilled water/phosphoric acid (99.95/0.05; v/v); mobile phase B: acetonitrile. The elution was performed with a gradient procedure as follows: 0-1 min, 2% B; 2–60 min, from 2% B to 98% B. The column heater was kept at 35°C. The flow rate used was 0.8 mL/min, and detection was performed at 210 nm. Each sample (10 *μ*L) was injected into the column after filtration through a 0.45 *μ*m filter disk. Identification of the tea polyphenols was carried out by comparing the retention times and the UV absorbance of the unknown peaks to those of the standards. A standard mixture containing theanine (Thea), theobromine (TB), caffeine (CAF), epigallocatechin (EGC), catechin (C), epicatechin (EC), epigallocatechin gallate (EGCG), gallocatechin (GC), gallocatechin gallate (GCG), and epicatechin gallate (ECG) in methanol was prepared and analyzed. Calibration curves for standard mixture was carried out using standard solutions injecting with volumes: 5, 10, and 20 *μ*L. The system was monitored by a computer equipped with the 32 Karat Software (Beckman Instrument Inc., Fullerton, CA, USA) for data collection, integration, and analysis.

### 2.3. Animals and Diets

Eight-week-old male C57BL/6 mice were obtained from the Laboratory Animal Services Centre (LASEC) of the Chinese University of Hong Kong. The care and use of the animals were in compliance with the institutional guidelines, and the experimental procedures were approved by the Animal Experimentation Ethics Committee of the CUHK. Animals were housed in standard cages (5 animals per cage) at a constant temperature of 20°C with a 12 h light/dark cycle. They were allowed *ad libitum* access to normal-chow diet and water. After 1 week of acclimatization, they were divided into six groups (*n* = 10 mice per group): (1) normal chow-fed group (N) that received normal chow diet containing 6% by weight of fat (SF04-057, Specialty Feeds, Glen Forest, Western Australia); (2) high-fat fed group (HF) that received a high-fat semipurified diet containing 21% wt/wt butterfat and 0.15% cholesterol (SF00-219, Specialty Feeds, Glen Forest, Western Australia); (3) high-fat fed group supplemented with 2% wt/wt green tea aqueous extract (HFLG); (4) high-fat fed group supplemented with 4% wt/wt green tea aqueous extract (HFHG); (5) high-fat fed group supplemented with 2% wt/wt cocoa tea aqueous extract (HFLC); and (6) high-fat fed group supplemented with 4% wt/wt cocoa tea aqueous extract (HFHC). The high-fat semipurified diet SF00-219 was formulated to be equivalent to western type diet Harlan Teklad TD88137. The normal chow diet SF04-057 was designed to be the control diet for the high-fat semipurified diet SF00-219. A detailed composition of SF00-219 and SF04-057 are summarised in Table 1. Food intake was recorded twice weekly, and body weights were measured twice a week.

### 2.4. Tissue Processing

Mice were fed diets for 8 weeks. At the end of the study, all mice were sacrificed after a 16-hour overnight fast. Animals were anaesthetised using a mixture of ketamine (100 mg/kg) and xylazine (10 mg/kg) i.p., and whole blood was withdrawn by cardiac puncture. Blood was collected using heparin containing tubes, and plasma was separated by centrifugation (3,000 rpm, 10 min). Plasma were used immediately for plasma lipid measurement or frozen (−80°C) for future use. Livers were immediately excised, weighed, and divided into smaller pieces for storage at −80°C (for lipid analysis) or in 4% paraformaldehyde for histological analysis. Epididymal, inguinal, and perirenal fat pads were excised and weighed. 

### 2.5. Biochemical Analyses

Plasma triglyceride (TG) and cholesterol (Chol) concentrations were measured by enzymatic methods, using GPO-PAP and CHOD-PAP kits (Roche Diagnostics, Switzerland), respectively. Total liver lipids were determined gravimetrically after extraction by the method of Bligh and Dyer [26]. Individual hepatic lipids were quantitated enzymatically (as described above) after solubilization in isopropanol.

### 2.6. Histology

 5 *μ*m thick sections of paraffin embedded tissue were cut with a microtome and placed on SuperFrost Plus microscope slides (Thermo Scientific, USA). Sections were dried at 37°C overnight and dewaxed by immersion with two exchanges in histolene (2 min) followed by rehydration to water through a series of graded ethanol washes comprising two washes in absolute ethanol, two washes in 95% ethanol, and two final washes in 75% ethanol. Sections were subjected to haematoxylin and eosin (H&E) staining, according to the method of Harris [27]. They were then dehydrated using graded ethanols comprising two washes in 75% ethanol, two washes in 95% ethanol, and two washes in absolute ethanol, after which they were subjected to two changes of xylene, and mounted with dibutyl phthalate in histolene (DPX) (Sigma-Aldrich, Inc., USA). Liver samples were examined histologically after embedding in paraffin, sectioning, and staining with H&E.

### 2.7. Gene Expression Analysis

Hepatic mRNA levels were measured by real-time PCR. Total RNA was isolated by selective binding to a silica gel-based membrane following the lysis and homogenisation of liver samples in a denaturing guanidine thiocyanate buffer (RNeasy kit, Qiagen, USA). RNA (100 ng) was reversely transcribed into cDNA using random primers provided with the iScript cDNA Synthesis kit (Bio-Rad, USA). Selected genes were amplified using iQ SYBR Green Supermix (Bio-Rad, USA) in an iCycler system (Bio-Rad, USA) with 12 pmol of both forward and reverse primers. PCR conditions were as follows: 1 cycle of 95°C for 3 min, 50 cycles of 95°C for 30 s, 55–60°C for 30 s and 72°C for 30 s, followed by 1 cycle of 95°C for 1 min. Purity of PCR products was assessed by melt curve analysis. Relative gene expression was calculated by normalizing cycle threshold (Ct) values for genes of interest with Ct values for glyceraldehyde-3-phosphate dehydrogenase (GAPDH) or cyclophilin using the delta-delta Ct method. Primer sequences were as follows: CYCLOPHILIN (forward: 5′-CAAATGCTGGACCAAACACAA-3′; reverse: 5′-CCATCCAGCCATTCAGTCTTG-3′); GAPDH (forward: 5′-GGCATCACTGCAACTCAGAA-3′; reverse: 5′-TTCAGCTCTGGGATGACCTT-3′); HMGCR (forward: 5′-CTTGTGGAATGCCTTGTGATTG-3′; reverse: 5′-AGCCGAAGCAGCACATGAT-3′); LDL-R (forward: 5′-CTGTGGGCTCCATAGGCTATCT-3′; reverse: 5′-GCGGTCCAGGGTCATCTTC-3′); PPAR-*γ* (forward: 5′-CCAGAGTCTGCTGATCTGCG-3′; reverse: 5′-GCCACCTCTTTGCTCTGCTC-3′); CD36 (forward: 5′-GAACCTATTGAAGGCTTACATCC-3′; reverse: 5′-CCCAGTCACTTGTGTTTTGAAC-3′).

### 2.8. Statistical Analysis

Values given in the text are means ± SEM, and Prism 5 for Window (version 5.0c, GraphPad Software, Inc., USA) was used for statistical analysis. Significant differences among all groups were assessed by one-way ANOVA, followed by Bonferroni's multiple comparison test. A probability of *P* < 0.05 was considered to be statistically significant.

## 3. Results

### 3.1. Chemical Profiles of Green Tea and Cocoa Tea Extracts

**Figure 1:**
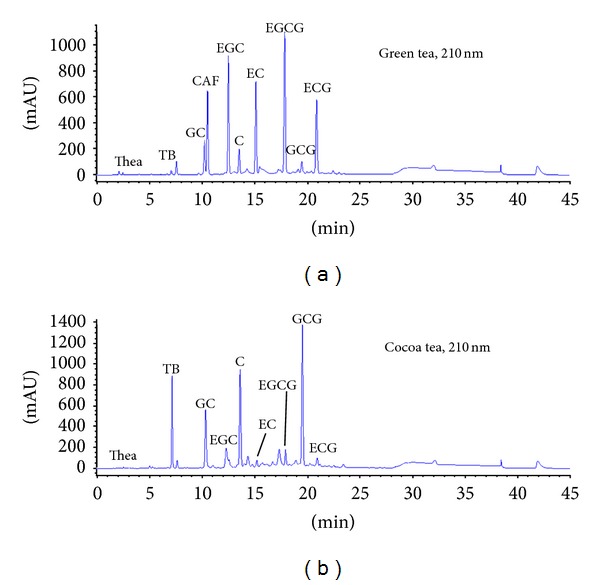
HPLC profiles of (a) green tea and (b) cocoa tea aqueous extracts. Detection was performed at UV 210 nm.

Figure 1 shows the HPLC profiles of (a) green tea and (b) cocoa tea aqueous extracts, respectively. The retention time point of the standards were compared to the HPLC profiles of both tea extracts, with the quantities for each of the chemical markers within the tea extracts calculated and summarized in Table 2. Cocoa tea possessed a chemical profile different from green tea, with a relative concentration of the six major tea catechins ranked GCG > C > EGCG > EGC > ECG > EC, whereas green tea extract had a relatively higher concentration of EGCG, followed by EGC, ECG, EC, C, and GCG. Cocoa tea extract also contained theanine and theobromine, with the content of 1.05 ± 0.09 and 10.32 ± 0.18%, respectively. Green tea extract contained a theanine composition of 1.59 ± 0.09% and a much lower contents of theobromine (0.35 ± 0.02%) compared to that of cocoa tea. The caffeine contents of green tea and cocoa tea extracts were 6.12 ± 0.03% and 0%, respectively. 

### 3.2. Effect of Green Tea and Cocoa Tea Supplementations on Daily Food Intake, Body Weight Gain, and Final Body Weight in Mice Given High-Fat Diet

Among all the high-fat-fed animals, addition of green tea or cocoa tea extract at 2% did not affect the daily food consumption of the animals. However, 4% green tea supplementation significantly reduced daily food consumption of C57BL/6 mice (2.6 ± 0.1 versus 3.0 ± 0.1 g, *P* < 0.01), whereby there was no significant difference on food intake for 4% cocoa tea supplemented group compared to HF group (Table 3). HF-fed animals gained significantly more weight than chow-fed animals (33.9 ± 1.3 versus 26.7 ± 0.8 g, *P* < 0.001). Both green tea and cocoa tea supplementation significantly reduced the final body weight and total body weight gain compared to mice given high-fat diet alone in a dose-dependent manner, as shown in Table 3. There was however no significant difference on final body weight or total weight gain between 2% green tea and cocoa tea supplemented groups or between 4% green tea and cocoa tea supplemented groups. 

### 3.3. Effect of Green Tea and Cocoa Tea Supplementations on Liver Weight and Liver to Body Weight Ratio in Mice Given High-Fat Diet

High-fat diet alone significantly increased liver weights of mice compared to mice given normal-chow diet. Mean liver weight was 35 ± 8% higher in the HF compared to the N group (*P* < 0.001). Both 2% and 4% green tea and cocoa tea supplementations had significant effect on liver weight in HF-fed mice (Table 4). When liver weights were expressed as a ratio relative to body wt, HF had a mean of 4.41 ± 0.16 g/100 g body wt, compared to the 4.34 ± 0.05 g/100 g for normal-chow-fed mice, which did not reach statistical significance. Both green tea and cocoa tea supplemented groups significantly reduced the liver to body weight ratio dose dependently compared to HF group. The % of reduction in liver/body weight ratio were 7.3 ± 3.4% and 11.9 ± 3.6% for 2% and 4% green tea supplementation and 12.3 ± 3.0% and 13.6 ± 2.6% for 2% and 4% cocoa tea supplementation, respectively. No significant difference was observed among all tea supplemented groups.

### 3.4. Effect of Green Tea and Cocoa Tea Supplementations on Epididymal, Perirenal, and Inguinal Fat Pad Weight in Mice Given High-Fat Diet

High-fat diet induced obesity in C57BL/6 male mice compared to normal-chow-fed mice after 8 weeks of diet, as evidenced by the significant increase in all three types of fat pad mass: epididymal fat mass (1467 ± 165 versus 397 ± 52 mg, *P* < 0.001), perirenal fat mass (517 ± 55 versus 139 ± 25 mg, *P* < 0.001), and inguinal fat mass (752 ± 135 versus 257 ± 52 mg, *P* < 0.001) (Table 5). Both green tea and cocoa tea supplementations significantly reduced all fat pad mass in a dose-dependent manner. There was no significant difference on epididymal fat mass or epididymal fat/body wt ratio and inguinal fat mass or inguinal fat/body weight ratio between 2% green tea and cocoa tea supplemented groups and likewise between 4% green tea and cocoa tea supplemented groups. However, perirenal fat mass tended to be lower for 2% green tea supplemented group compared to 2% cocoa tea supplemented group and was significantly lower when expressed as perirenal fat wt/body wt ratio. There was however no significant difference on perirenal fat mass and perirenal fat wt/body wt ratio between 4% green tea and cocoa tea supplemented groups (Table 5). 

### 3.5. Effect of Green Tea and Cocoa Tea Supplementations on Liver Histology and Liver Lipid Contents in Mice Given High-Fat Diet

**Figure 2:**
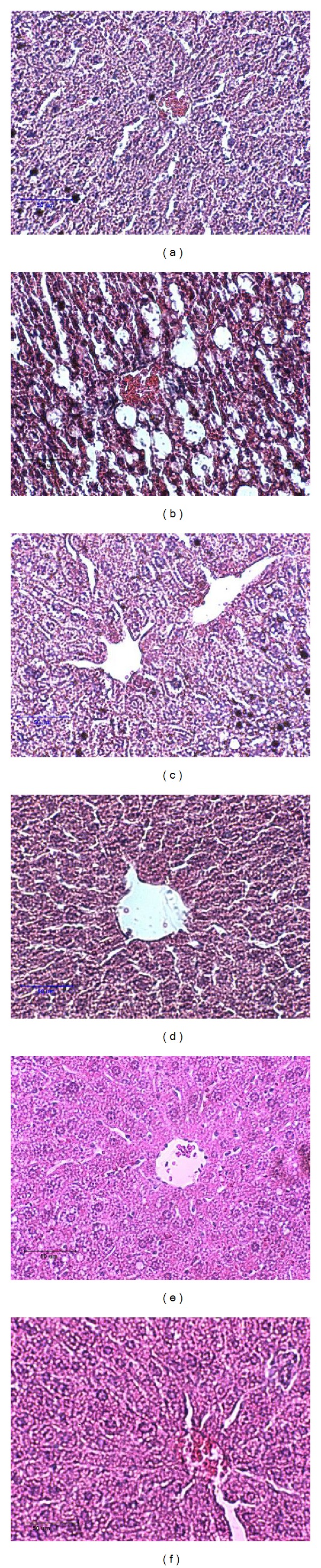
Histological appearance of liver sections of (a) a normal chow-fed mice; (b) a high-fat fed mice; (c) a high-fat fed mice supplemented with 2% green tea extract; (d) a high-fat fed mice supplemented with 4% green tea extract; (e) a high-fat fed mice supplemented with 2% cocoa tea extract; and (f) a high-fat fed mice supplemented with 4% cocoa tea extract. Sections were stained with haematoxylin and eosin. Lipid accumulation in the liver of HF-fed mice was very evident due to the presence of circular lipid droplets. Circular lipid droplets were significantly reduced in sections from both green tea and cocoa tea supplemented animals.

Livers from mice given different diets were analyzed histologically, and representative stained sections are shown in Figure 2. N group animals demonstrated the histological sections of normal livers. In contrast, H&E sections from HF animals revealed the presence of a large number of circular lipid droplets in between hepatocytes. These lipid inclusions were clearly reduced in both size and number in livers of both doses of green tea- and cocoa tea-treated animals.

**Figure 3:**
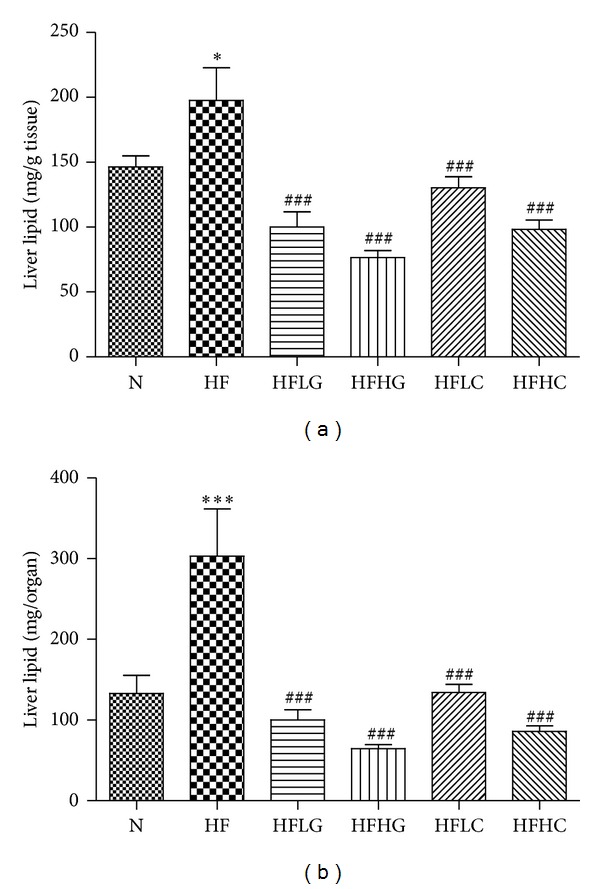
Total lipid in the liver of mice fed a normal chow or high-fat diet, with or without the addition of green tea or cocoa tea supplementation at different concentrations. (a) shows results obtained gravimetrically expressed as mg of lipid per grams of liver; (b) shows results expressed as mg of lipid per whole liver. Mice were fed diets for 8 weeks. Values represent means ± SEM (*n* = 10). Significant difference between N and HF mice using one-way ANOVA: ***P < 0.001. Significant difference among HF and HFLG, HFHG, HFLC, and HFHC mice using one-way ANOVA: ^###^ *P* < 0.001.

**Figure 4:**
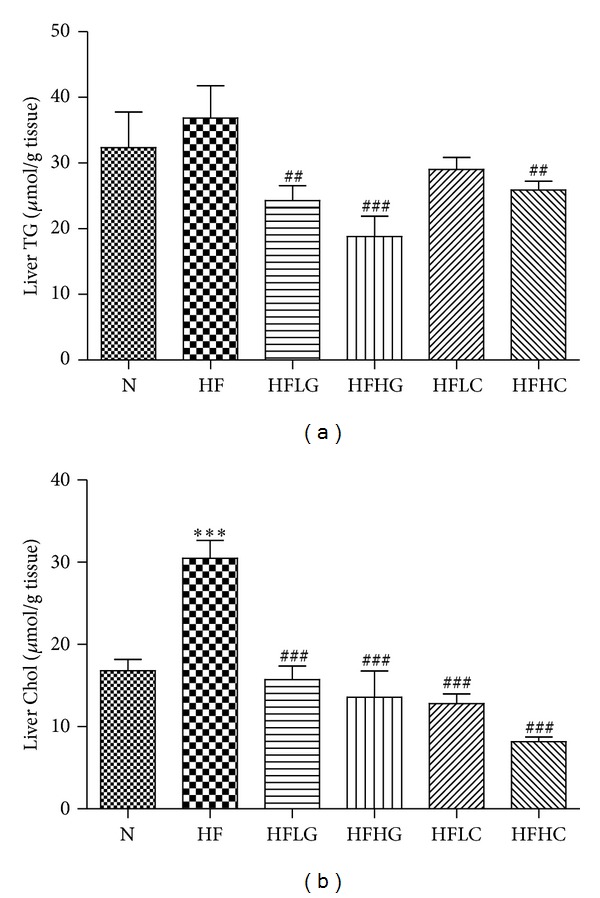
Liver TG (a) and Chol (b) of mice fed a normal chow or high-fat diet, with or without the addition of green tea or cocoa tea supplementation at different concentrations. Results expressed as *μ*mol/grams of liver. Mice were fed diets for 8 weeks. Values represent means ± SEM (*n* = 10). Significant difference between N and HF mice using one-way ANOVA: ***P < 0.001. Significant difference among HF and HFLG, HFHG, HFLC, and HFHC mice using one-way ANOVA: ^##^ *P* < 0.01, ^###^ *P* < 0.001.

 The beneficial effect of both tea supplementations on HF-induced hepatomegaly was associated with a significant reduction in total hepatic lipid content, expressed as mg of lipid per grams of liver (Figure 3(a)) or expressed as mg of lipid per whole liver (Figure 3(b)). Total liver lipid content (in mg/g tissue) was significantly higher in HF mice compared to N mice (32 ± 11% higher, *P* < 0.05). HFLG-, HFHG-, HFLC-, and HFHC-fed mice had liver lipid levels that were significantly lower than HF-fed mice (i.e., 47 ± 4%, 56 ± 6%, 26 ± 6%, and 47 ± 4%, resp., for mg/g tissue). No significant difference was observed among green tea and cocoa tea supplemented groups at either dose. As shown in Figure 4, measurement of liver TG (a) and Chol (b) revealed that HF mice had elevated levels of both lipids compared to N mice (i.e., 1.2-fold (±0.1) increase in TG and 1.9-fold (±0.1) increase in Chol), of which only the latter had reached statistical significance. 2% and 4% green tea supplementation significantly reduced liver TG and Chol levels in HF-fed mice. Cocoa tea supplementation, on the other hand significantly reduced liver Chol at both 2% and 4% (55.9 ± 3.2%, *P* < 0.001, and 72.1 ± 1.6%, *P* < 0.001). Although both 2% and 4% cocoa tea additions resulted in reduced levels of liver TG (11.5 ± 5.5% and 20.8 ± 4.9%, *P* < 0.01), only 4% supplementation reached statistical significance.

### 3.6. Effect of Green Tea and Cocoa Tea Supplementations on Plasma Lipid in Mice Given High-Fat Diet

Plasma lipid levels of mice are shown in Table 6. Compared to N-fed mice, HF mice had significantly elevated levels of plasma TG and Chol. Both green tea and cocoa tea additions resulted in significant reduced levels of plasma TG and Chol at either dose. However, there was no significant difference on TG or Chol between 2% green tea and cocoa tea supplemented groups or between 4% green tea and cocoa tea supplemented groups. 

### 3.7. Effect of Green Tea and Cocoa Tea Supplementations on Hepatic Gene Expression in Mice Given High-Fat Diet

**Figure 5:**
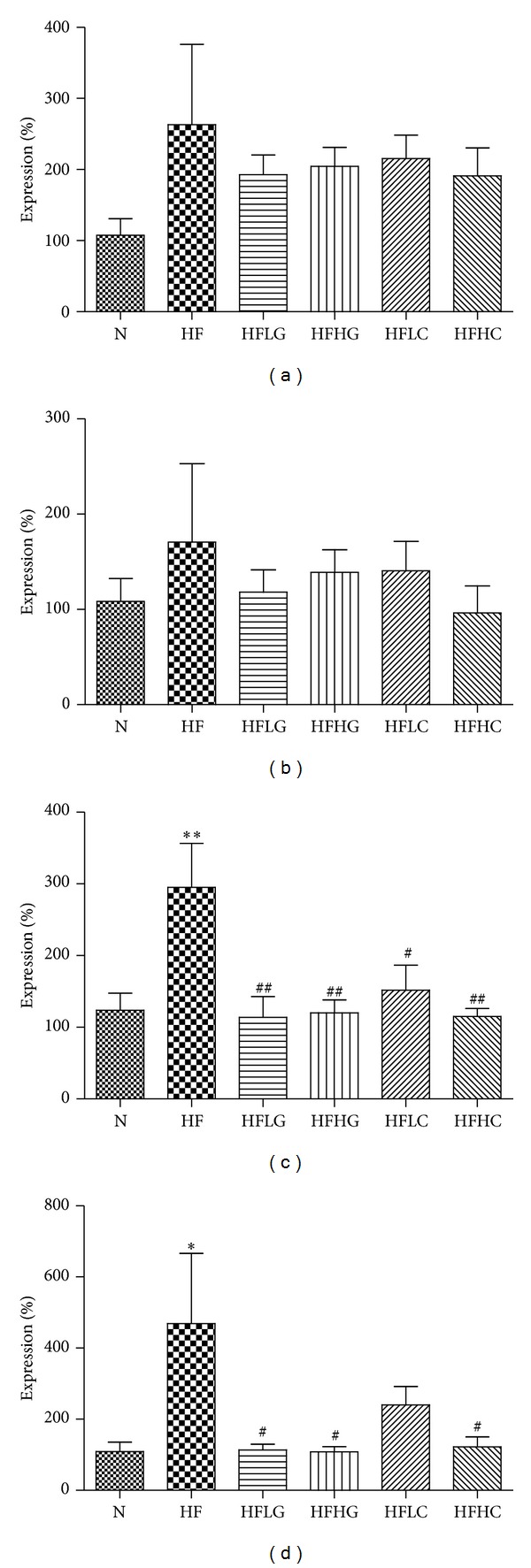
Effect of green tea and cocoa tea supplementations on (a) LDL-R; (b) HMG Co-A reductase; (c) CD36; and (d) PPAR-*γ* mRNA levels in the liver of chow-fed and high-fat-fed mice. Mice were fed diets for 8 weeks. Values represent means ± SEM (*n* = 3-4). Significant difference between N and HF mice using one-way ANOVA: *P < 0.05, **P < 0.01. Significant difference among HF and HFLG, HFHG, HFLC, and HFHC mice using one-way ANOVA: ^#^ *P* < 0.05, ^##^ *P* < 0.01.

In order to shed light on the mechanism by which cocoa tea extract contributes to its beneficial effects, mRNA levels of proteins controlling lipid metabolism in the liver were determined (Figure 5). Relative mRNA levels were compared by expressing levels relative to N mice. High-fat diet tended to increase level of expression of genes controlling cholesterol uptake—LDL-R, and cholesterol synthesis—HMG Co-A reductase, which were tended to be lowered by green tea and cocoa tea supplementations. mRNA levels for genes involved in fatty acid uptake, CD36 was also higher in HF group. Likewise, green tea and cocoa tea supplementations dose-dependently reduced the level of expression. mRNA levels for the transcription factor controlling lipid metabolism, PPAR-*γ* was markedly increased by high-fat diet. Both green tea and cocoa tea supplementations dose-dependently downregulated the expression at 2% and 4%, however only 2% and 4% green tea and 4% cocoa tea supplementation had reached statistical significance. 

## 4. Discussion

The present study demonstrates that the addition of cocoa tea to mice on a high-fat diet (containing 21% butterfat and 0.15% cholesterol) resulted in a dose-dependent reduction in: (a) body weight, (b) fat pad mass (epididymal, perirenal and inguinal fat), (c) liver weight, (d) total liver lipid, (e) liver triglyceride and cholesterol, and (f) plasma lipid levels. These results provided evidence for the first time indicating that cocoa tea has a potential beneficial effect on weight control and lipid metabolism in C57BL/6 mice with diet-induced obesity. We are also the first time comparing the effects of cocoa tea and green tea using a diet-induced mice model which mimics human with metabolic syndrome. 

Over the years, the beneficial effects of dietary green tea on plasma and hepatic lipid levels have been well documented in various animal studies [8, 9, 28, 29]. The positive findings from these pre-clinical studies have also been confirmed in a number of human clinical trials suggesting the antiobesity effect of green tea extract [7, 30, 31]. The beneficial effects of green tea have been attributed to the presence of tea catechins, in particularly EGCG [32–34]. Our data pertaining to the catechins contents of both tea extracts (Table 2) indicate that the major type of catechins in green tea extract was EGCG. This was however not the case for cocoa tea extract, which contained only approximately one-seventh the amount of EGCG detected in green tea extract, but with higher quantities of other catechins such as GCG and C. Previous work using GCG produced by heat epimerization of EGCG suggested GCG are more effective in lowering plasma cholesterol concentration compared to EGCG, possibly because GCG are more effective in inhibiting lymphatic absorption of endogenous cholesterol [35–37], but not triglyceride [36]. In consonance with these studies, when comparing the individual Chol and TG lowering effects of green tea and cocoa tea in our animal experiment, it appeared that green tea extract are more potent in lowering both plasma and hepatic TG, whereby cocoa tea extract are comparatively more potent in lowering plasma and hepatic Chol. The above studies therefore support the notion that GCG has played a role in contributing the beneficial effects of cocoa tea. 

The preceding discussion assumes that the lipid-lowering and hepatoprotective properties of cocoa tea extract are dependent on its catechins content and/or its distinct catechin composition. It cannot be ruled out however, that the noncatechins containing portion of cocoa tea extract may also be responsible for the observed effects. HPLC analysis suggested that cocoa tea extract also contained high levels of theobromine. Although researches focusing on the effect of theobromine on lipid metabolism are limited, Eteng and Ettarh showed that acute theobromine administration (700 mg/kg) caused significant reduction in total serum cholesterol, and triglycerides [38]. Only additional studies with fractionated cocoa tea will provide the necessary evidence as to determine the bioactive component of cocoa tea extract.

A number of different mechanisms could be responsible for the beneficial effects of cocoa tea on obesity and plasma and liver lipid metabolism. It had been suggested that green tea catechins could exert modifications in appetite control, leading to decreased nutrient absorption and thereby contributing to a downregulation of enzymes involved in lipid metabolism within the liver and adipose tissues [39, 40]. In the present study, 4% green tea supplemented animals had significantly reduced appetite, as evidenced by the reduced food intake compared to high-fat-fed animals. This was however not the case for cocoa tea supplemented animals. Cocoa tea supplementation dose-dependently reduced the body weight and plasma and hepatic lipid without affecting the food intake of the animals. Furthermore, this reduction in hepatic cholesterol levels induced by cocoa tea extract was associated with a trend in the reduction in the expression of enzymes affecting cholesterol uptake (LDL-R) and cholesterol synthesis (HMG Co-A reductase). Cocoa tea could also downregulate the expression of enzyme affecting fatty acid uptake (CD36), thereby leading to its TG-lowering effect. Interestingly, cocoa tea could reduce high-fat diet-induced increase in PPAR-*γ* expression. PPAR-*γ* is a ligand-activated transcription factor that played an important role in the regulation of lipid and glucose metabolisms. It regulates a serial of genes involved in lipid metabolism including SCD-1, CD36, FAS, LDL-R, and SREBP-1. In rodent models of metabolic syndrome demonstrating diet-induced hepatic steatosis and obesity, hepatic expression of PPAR*γ* is markedly upregulated [41, 42]. The fact that the elevated transcription levels of PPAR*γ* and its targeted genes including LDL-R, HMG Co-A reductase and CD36 in the livers of high-fat-fed mice are dose dependently downregulated in cocoa tea supplemented animals suggested cocoa tea could possibly regulate hepatic lipid metabolism via PPAR-*γ* regulated pathway. Kurihara et al. suggested that cocoa tea reduced blood TG via suppression of lymphatic TG absorption [25]. Whether the effect of cocoa tea supplementation on body weight and downregulation of plasma and liver lipid observed in our study was due to the ability of cocoa tea to primarily regulate hepatic PPAR-*γ* expression and its targeted genes or secondary as a result of reduced lipid absorption within the intestine, the present study has the limitation in providing direct evidence of reduced lipid absorption and/or reduced bile acid production. Additional experiments including gene and protein expression analysis within the liver, adipose tissues, and intestine will be required to define which of these effects of cocoa tea are primary and secondary.

The potent ability of dietary cocoa tea to reduce both plasma and liver lipid content in high-fat-fed mice, to an extent similar to green tea of the same dosage, suggests it might be of therapeutic benefit in humans with diet-induced metabolic syndrome, in particularly for obese individuals with hyperlipidemia and nonalcoholic fatty liver disease (NAFLD). NAFLD affects 10–20% of the general population and is commonly found in obese or diabetic patients [43]. At present, there is no established treatment for it, and current suggested management strategy relies on diet regimen, weight loss, and exercise. Dietary supplements/nutraceuticals that might help to delay the development or alleviate this condition are therefore of great importance [44, 45]. Although green tea promotes beneficial effect on health, the fact that green tea contains caffeine has caused various concerns. Caffeine can cause insomnia, anxiety, irritability, upset stomach, nausea, diarrhea, or frequent urination [46] and has been reported to cause interact with various drugs [47]. It is generally recommended that green tea should not be taken by patients suffering from heart conditions or major cardiovascular problems [9, 48]. Pregnant and breastfeeding women and children are suggested not to drink more than one or two cups per day due to their slow detoxification rate of caffeine [8, 19]. Current green tea decaffeination technique predominately relies on the use of supercritical carbon dioxide fluid extraction (SC-CO_2_) method. Although this method has great advantages over conventional methods which employ the use of solvents that could be toxic [19], SC-CO_2_ are relatively cost ineffective and require high capital costs for batch extraction [49]. During the decaffeination process, much of the volatile and aroma-active compounds in the green tea will also be removed, thereby resulting in weaker aroma and stronger bitter taste [19, 50]. The fact that cocoa tea, being a naturally occurring tea plant, contains no caffeine therefore poses major advantage. This unique feature of cocoa tea also provides an opportunity for the more feasible through-the-day consumption, so that it could be integrated with the daily dietary regimen of obese individuals and subjects with cardiovascular problems, without the excitatory effects of caffeine. Further studies will help to determine whether cocoa tea is of therapeutic benefit in patients with NAFLD or in overweight and insulin-resistant individuals at increased risk of coronary artery disease.

In conclusion, the present study indicates that cocoa tea has a beneficial effect on obesity, hepatomegaly, hepatic steatosis, and elevated plasma lipid levels in mice fed with a high-fat diet. These results provide the proof of concept that cocoa tea, which contains no caffeine, might be of therapeutic value as a nutraceutical in patients with increased risk of fatty liver disease and/or cardiovascular disease. Furthermore, the unique feature of the naturally decaffeinated cocoa tea has posed major advantage over green tea or decaffeinated green tea in providing individuals whom are prohibited from caffeine intake with the feasibility and flexibility to enjoy the benefits of tea, without the concern of the side effects induced by caffeine or the high cost incurred during the processing of decaffeinated green tea. 

## Figures and Tables

**Table 1:** Ingredient composition of normal control diet, SF04-057, and high-fat semipurified diet, SF00-219.

Ingredients	SF04-057 (g/kg)	SF00-219 (g/kg)
Casein (acid)	195	195
Sucrose	341	341
Canola oil	60	0
Clarified Butter (ghee)	0	210
Cellulose	50	50
Wheat starch	306	154
DL methionine	3.0	3.0
Calcium carbonate	17.1	17.1
Sodium chloride	2.6	2.6
AIN93 trace minerals	1.4	1.4
Potassium citrate	2.5	2.6
Potassium dihydrogen phosphate	6.9	6.9
Potassium sulphate	1.6	1.6
Choline chloride (60%)	2.5	2.5
SF00-219 vitamins	10	10
Cholesterol	0	1.5
Oxicap E2	0.04	0.04

**Table 2:** Amount of each chemical markers within cocoa tea and green tea extracts.

Chemical markers	Quantity (mg/100 mg)	
	Green tea	Cocoa tea
Thea	1.59 ± 0.19	1.05 ± 0.09
TB	0.35 ± 0.02	10.32 ± 0.18
CAF	6.12 ± 0.03	Not detected
EGC	5.57 ± 0.04	1.03 ± 0.14
C	1.43 ± 0.07	7.44 ± 0.11
EC	4.95 ± 0.48	0.44 ± 0.01
EGCG	8.54 ± 0.09	1.17 ± 0.08
GCG	0.58 ± 0.01	11.07 ± 0.18
ECG	5.17 ± 0.04	0.68 ± 0.02

Values represent means ± SEM (*n* = 3).

**Table 3:** Body weights and daily food intake of mice fed normal chow or a high-fat diet with or without green tea or cocoa tea supplementation at different concentrations.

	N	HF	HFLG	HFHG	HFLC	HFHC
Initial body wt. (g)	22.7 ± 0.4	23.1 ± 0.3	22.8 ± 0.6	22.5 ± 0.6	22.7 ± 0.6	22.5 ± 0.5
Final body wt. (g)	26.7 ± 0.8	33.9 ± 1.3***	26.4 ± 0.7^###^	23.8 ± 0.7 ^###^	28.5 ± 0.5^###^	25.6 ± 0.4^###^
Wt. gain (g)	4.0 ± 0.5	10.8 ± 1.1***	3.6 ± 0.4^###^	1.3 ± 0.3^###^	5.9 ± 0.4^###^	3.1 ± 0.3^###^
Daily food intake (g)	2.6 ± 0.1	3.0 ± 0.1	2.8 ± 0.1	2.6 ± 0.1^##^	2.8 ± 0.1	2.7 ± 0.1

Values represent means ± SEM (*n* = 10).

Significant difference between N and HF mice using one-way ANOVA: ***P < 0.001.

Significant difference between HF and HFLG, HFHG, HFLC, and HFHC mice using one-way ANOVA: ^##^ *P *< 0.01, ^###^ *P* < 0.001.

**Table 4:** Liver weights and liver to body weight ratio of mice fed normal chow or a high-fat diet with or without green tea or cocoa tea supplementation at different concentrations.

	N	HF	HFLG	HFHG	HFLC	HFHC
Liver wt. (g)	1.08 ± 0.04	1.43 ± 0.10***	0.99 ± 0.03^###^	0.84 ± 0.03^###^	1.02 ± 0.02^###^	0.87 ± 0.02^###^
Liver wt./body wt. (g/100 g)	4.34 ± 0.05	4.41 ± 0.16	4.05 ± 0.05^#^	3.83 ± 0.04^###^	3.83 ± 0.04^###^	3.77 ± 0.03^###^

Values represent means ± SEM (*n* = 10).

Significant difference between N and HF mice using one-way ANOVA: ***P < 0.001.

Significant difference between HF and HFLG, HFHG, HFLC, and HFHC mice using one-way ANOVA: ^#^ *P* < 0.05, ^###^ *P* < 0.001.

**Table 5:** Fat pad weights of mice fed normal chow or a high-fat diet with or without green tea or cocoa tea supplementation at different concentrations.

	N	HF	HFLG	HFHG	HFLC	HFHC
Epididymal fat pad wt. (mg)	397 ± 52	1467 ± 165***	442 ± 64^###^	233 ± 19^###^	632 ± 50^###^	329 ± 31 ^###^
Epididymal fat pad wt./body wt. (g/100 g)	1.55 ± 0.17	4.46 ± 0.37 ***	1.77 ± 0.21^###^	1.07 ± 0.08^###^	2.35 ± 0.16^###^	1.42 ± 12^###^
Perirenal fat pad wt. (g)	139 ± 25	517 ± 55***	135 ± 19^###^	67 ± 6^###^	232 ± 20 ^###^	88 ± 12^###^
Perirenal fat pad wt./body wt. (g/100 g)	0.54 ± 0.08	1.58 ± 0.13***	0.54 ± 0.06^###^	0.31 ± 0.03^### ^	0.86 ± 0.07^###+^	0.38 ± 0.05^###^
Inguinal fat pad wt. (g)	257 ± 52	752 ± 135***	258 ± 41^###^	205 ± 10^###^	394 ± 32^##^	214 ± 20^###^
Inguinal fat pad wt./body wt. (g/100 g)	0.99 ± 0.17	2.25 ± 0.34***	1.03 ± 0.14^### ^	0.95 ± 0.06^###^	1.47 ± 0.10^# ^	0.92 ± 0.08^###^

Values represent means ± SEM (*n* = 10).

Significant difference between N and HF mice using one-way ANOVA: ***P < 0.001.

Significant difference between HF and HFLG, HFHG, HFLC, and HFHC mice using one-way ANOVA: ^#^ *P* < 0.05, ^##^ *P* < 0.01, ^###^ *P* < 0.001.

Significant difference between HFLG and HFLC mice using one-way ANOVA: ^+^ *P* < 0.05.

**Table 6:** Plasma lipid in mice fed normal chow or a high-fat diet with or without green tea or cocoa tea supplementation at different concentrations.

	N	HF	HFLG	HFHG	HFLC	HFHC
Triglyceride (mmol/L)	0.92 ± 0.02	1.15 ± 0.05*	0.57 ± 0.02^###^	0.49 ± 0.04^###^	0.92 ± 0.02^#^	0.65 ± 0.02^###^
Cholesterol (mmol/L)	2.72 ± 0.13	5.89 ± 0.20^∗∗∗^	3.68 ± 0.09^###^	3.54 ± 0.13^###^	3.67 ± 0.07^###^	3.33 ± 0.10^###^

Values represent means ± SEM (*n* = 10).

Significant difference between N and HF mice using one-way ANOVA: *P < 0.05, ***P < 0.001.

Significant difference among HF and HFLG, HFHG, HFLC, and HFHC mice using one-way ANOVA: ^#^ *P* < 0.05, ^###^ *P* < 0.001.
